# Ultrasound-Guided, Single-Entry, Dual-Target Hydrodissection for Carpal Tunnel Syndrome: A Technical Note With Cadaveric Validation

**DOI:** 10.7759/cureus.107717

**Published:** 2026-04-25

**Authors:** Anwar Suhaimi, Yonghyun Yoon, Teinny Suryadi, Jaeyoung Lee, Jaewoo Lim, Jungyoun Kim, Jihyo Hwang, Sang-Hyun Kim, U-Young Lee, King Hei Stanley Lam

**Affiliations:** 1 Rehabilitation Medicine, University Malaya Medical Centre, Kuala Lumpur, MYS; 2 Rehabilitation Medicine, University Malaya, Kuala Lumpur, MYS; 3 Orthopaedics, International Academy of Musculoskeletal Medicine, Hong Kong, HKG; 4 Orthopaedics, International Academy of Regenerative Medicine, Incheon, KOR; 5 Orthopaedics, MSKUS, San Diego, USA; 6 Orthopaedic Surgery, Hallym University Kangnam Sacred Heart Hospital, Seoul, KOR; 7 Orthopaedic Surgery, Incheon Terminal Orthopaedic Surgery Clinic, Incheon, KOR; 8 Department of Physical Medicine and Rehabilitation, Medistra Hospital, Jakarta, IDN; 9 Physical Medicine and Rehabilitation, Synergy Clinic, Jakarta, IDN; 10 Department of Physical Medicine and Rehabilitation, Hermina Podomoro Hospital, Jakarta, IDN; 11 Orthopaedics, Incheon Terminal Orthopaedic Surgery Clinic, Incheon, KOR; 12 Department of Orthopaedic Surgery, Hallym University Kangnam Sacred Heart Hospital, Seoul, KOR; 13 Anatomy, College of Korean Medicine, Woosuk University, Jeonju, KOR; 14 Department of Anatomy, Catholic Institute for Applied Anatomy, College of Medicine, Catholic University of Korea, Seoul, KOR; 15 Faculty of Medicine, The Chinese University of Hong Kong, New Territories, HKG; 16 Faculty of Medicine, The University of Hong Kong, Hong Kong, HKG; 17 The Board of Clinical Research, The Hong Kong Institute of Musculoskeletal Medicine, Kowloon, HKG

**Keywords:** cadaveric validation, carpal tunnel syndrome, hydrodissection, median nerve, minimally invasive procedure, peripheral nerve entrapment, transverse carpal ligament, ultrasound-guided injection

## Abstract

Ultrasound-guided hydrodissection (HD) is an emerging minimally invasive treatment for carpal tunnel syndrome (CTS); however, achieving circumferential perineural spread around the median nerve may require repeated needle repositioning, multiple skin entries, or combined in-plane and out-of-plane maneuvers, thereby increasing procedural complexity. This technical report describes a novel ultrasound-guided single-entry dual-target HD technique for CTS that sequentially addresses the undersurface of the transverse carpal ligament (TCL) and the perineural plane surrounding the median nerve through a standardized scanning approach initiated at the first carpometacarpal (1st CMC) joint and maintained during medial transducer translation across the carpal tunnel. The technique uses a structured in-plane distal-to-proximal trajectory to maintain continuous needle visualization while reaching both targets through a single skin entry. Importantly, the approach targets the deep surface of the TCL but does not constitute TCL release.

For cadaveric validation, the same procedural approach was performed in one fresh-frozen cadaver using both wrists. Under ultrasound guidance, 10 mL of methylene blue was injected per wrist using a 23-gauge, 6-cm needle, followed by dissection. Cadaveric dissection demonstrated dye surrounding the median nerve on both its superficial and deep aspects, supporting circumferential perineural halo formation, with proximal longitudinal spread from the entry site. These findings support the anatomical plausibility of the intended HD plane. This structured single-entry dual-target approach may offer a practical technical framework for CTS hydrodissection; however, further prospective and comparative studies are required to determine its clinical efficacy, safety, and relative advantages over existing ultrasound-guided CTS techniques.

## Introduction

Median nerve entrapment at the carpal tunnel is a common disorder associated with substantial functional impairment and a significant socioeconomic burden. Ultrasound-guided hydrodissection (HD) has improved the precision and safety of minimally invasive treatments for carpal tunnel syndrome (CTS). However, many currently described techniques primarily emphasize either perineural HD of the median nerve or ligament-focused interventions at the transverse carpal ligament (TCL), rather than addressing both components in a coordinated manner [1-3].

In addition, achieving circumferential perineural spread around the median nerve may require repeated needle repositioning, multiple skin entries, or a combination of in-plane and out-of-plane maneuvers [2,4,5]. Although such approaches may be effective, they can increase procedural complexity and technical demands. Emerging evidence suggests that CTS is not simply a disorder of focal nerve compression, but rather a multifactorial entrapment neuropathy involving TCL-related mechanical constraints, subsynovial fibrosis, perineural adhesions, and impaired median nerve gliding [1,3,4]. From this perspective, an ultrasound-guided intervention may be designed to address both the deep surface of the TCL and the perineural plane surrounding the median nerve in a sequential manner. Importantly, HD along the undersurface of the TCL in this context is intended to establish and expand the working plane beneath the ligament and does not constitute a TCL release.

We describe a novel ultrasound-guided, single-entry, dual-target HD technique for CTS using a standardized scanning approach initiated at the first carpometacarpal (1st CMC) joint and maintained during medial transducer translation across the carpal tunnel. This workflow sequentially addresses the TCL undersurface and the perineural plane surrounding the median nerve through a single oblique distal-to-proximal in-plane trajectory.

## Technical report

Indications and precautions

Appropriate clinical indications, major contraindications, and key procedural precautions for the proposed technique are summarized in Table 1.

**Table 1:** Indications, contraindications, and key precautions for the proposed technique CTS: carpal tunnel syndrome

Category	Details
Indications	Mild-to-moderate CTS supported by clinical and electrodiagnostic findings, without evidence of axonal loss or denervation
	Persistent or recurrent symptoms despite conservative treatment for at least 6-12 weeks
	Sonographic features supportive of entrapment, such as proximal median nerve swelling or increased median nerve cross-sectional area
Contraindications	Local infection at the planned injection site
	Known allergy to the intended injectate
	Inability to cooperate with the procedure
	Suspected space-occupying lesion requiring alternative diagnostic or therapeutic management
Key precautions	Anticoagulation or elevated bleeding risk
	Anatomical variants, including a bifid median nerve or a persistent median artery
	Marked tenosynovitis or distorted local anatomy requiring modification of the needle trajectory
	Severe CTS with marked thenar weakness/atrophy or electrodiagnostic evidence of axonal loss may warrant surgical evaluation rather than injection alone

Equipment and patient positioning

A high-frequency linear ultrasound transducer (12-18 MHz) is used. Needle selection for the present technique consists of a 23-gauge, 6-cm needle. For the clinical procedure, 5% dextrose in water (D5W) is used as the injectate, administered in small aliquots to facilitate progressive HD. No local anesthetic is used at the skin entry site [4]. The patient is positioned supine with the arm abducted, the forearm supinated, and the wrist maintained in a neutral or slightly extended position. The operator and ultrasound monitor are aligned to optimize ergonomics and to maintain continuous in-plane needle visualization throughout the procedure.

Scanning protocol and sonographic landmarks

A structured scanning sequence is used to improve anatomical orientation and procedural consistency. The scan begins at the level of the first carpometacarpal (1st CMC) joint, with the cortical surface of the first metacarpal maintained in plane. As the transducer is translated medially while maintaining the initial 1st CMC plane, the osseous cortical landmark disappears and is replaced by a transitional level in which the TCL attachment remains visible and the carpal tunnel contents come into view. With further ulnar translation, the pisiform is identified while maintaining visualization of the tunnel contents.

**Figure 1:**
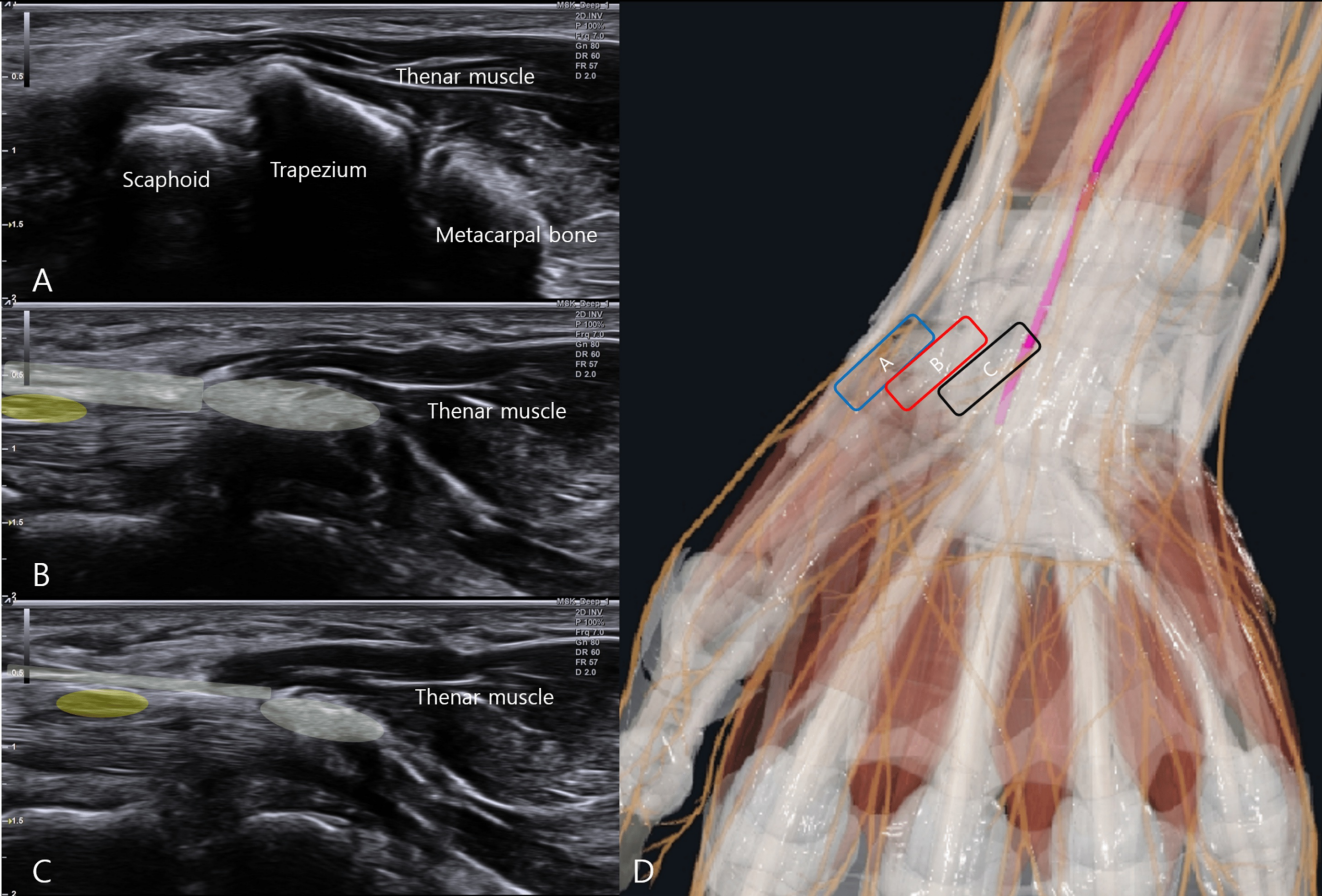
Standardized sonographic landmarking for the 1st CMC-initiated medial translation scanning approach (A) Distal starting level at the first carpometacarpal (1st CMC) joint, where the cortical surface of the first metacarpal is maintained in plane. (B) Transitional level reached during medial transducer translation after disappearance of the initial bony cortical landmark, where the transverse carpal ligament (TCL; green shading) remains visible, and the carpal tunnel contents begin to come into view. (C) Intended injection level demonstrating the TCL (green shading), the median nerve (yellow shading), and the adjacent flexor tendons within the maintained scanning plane. (D) Schematic illustration of the probe positions corresponding to panels A-C, together with the maintained scanning corridor during medial transducer translation and the planned single-entry oblique distal-to-proximal in-plane working pathway

The scaphoid- and pisiform-sided margins of the transverse carpal ligament may be appreciated during this medial translation. Still, the working plane in the present technique is established by maintaining the initial 1st CMC plane rather than by defining a separate scaphoid-to-pisiform scanning plane. Needle advancement is then performed within this maintained plane using an oblique distal-to-proximal in-plane trajectory. Before needle insertion, a mandatory safety scan is performed to identify vascular variants, a bifid median nerve, a persistent median artery, tenosynovitis, or space-occupying lesions such as ganglia. The key sonographic landmarks, intended target structures (the transverse carpal ligament and median nerve), injection level, and probe orientation are shown in Figure 1.

Single-entry, dual-target HD technique

**Figure 2:**
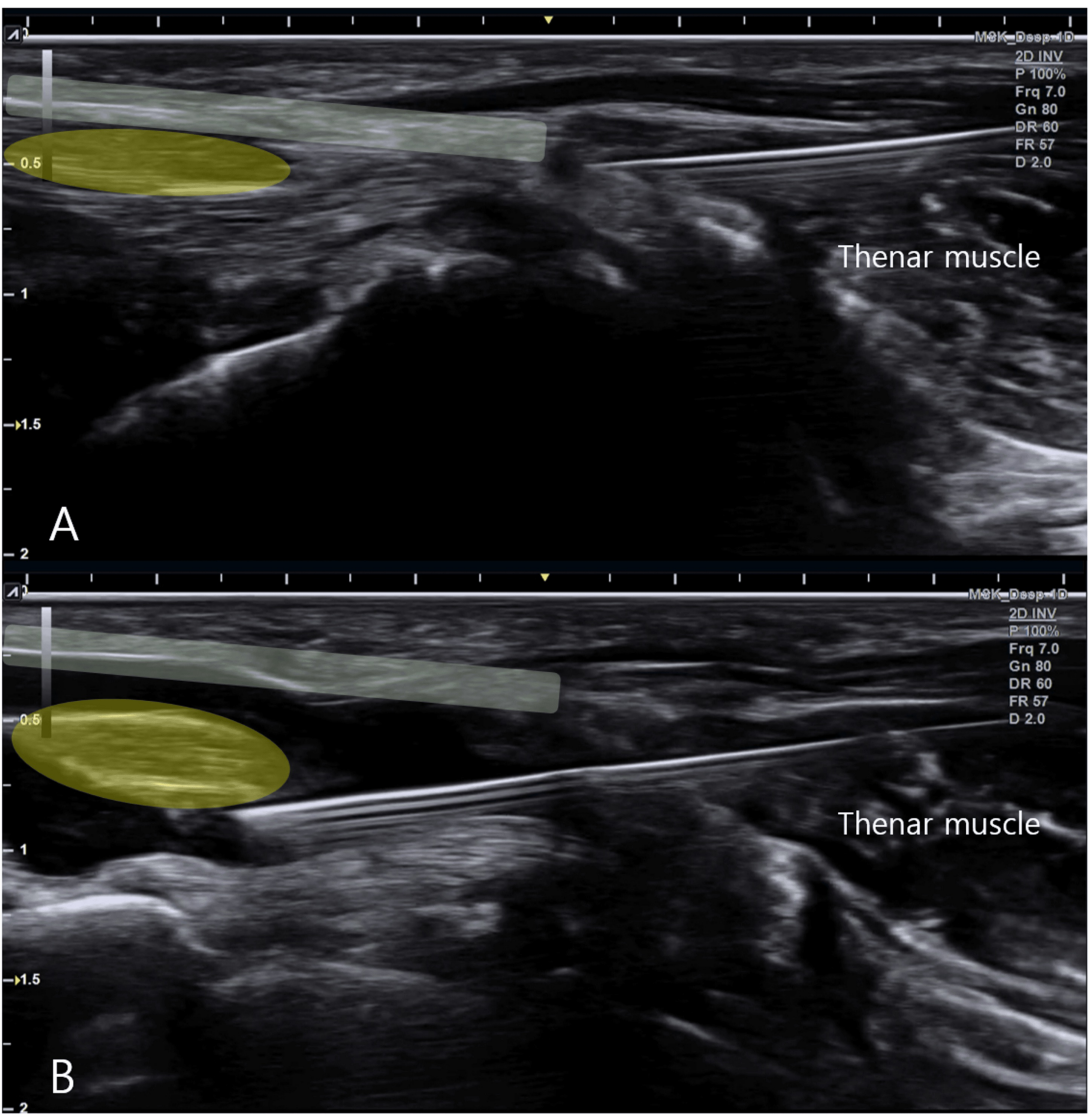
Real-time ultrasound-guided single-entry dual-target hydrodissection technique After tracing from the first carpometacarpal (1st CMC) starting level and maintaining the same scanning plane during medial transducer translation, the needle is advanced in plane along an oblique distal-to-proximal trajectory. In both panels, the transverse carpal ligament (TCL) is highlighted in green and the median nerve in yellow (A) Pre-injection image at the intended hydrodissection level, demonstrating the median nerve beneath the TCL before fluid separation. (B) Hydrodissection image demonstrating injectate spread beneath the TCL and around the median nerve through the same single-entry pathway, consistent with circumferential perineural halo formation

A single skin entry point is selected to permit uninterrupted in-plane advancement along the maintained 1st CMC-based scanning plane established during medial transducer translation across the carpal tunnel. The entry point is positioned just medial to the scaphoid and trapezium tubercles, where the palmar cutaneous branch is less likely to traverse the intended working corridor and where the TCL attachment site can be approached along an anatomically favorable trajectory. Needle entry is performed approximately one fingerbreadth from the probe, with the bevel initially oriented upward. The needle is then advanced in plane along an oblique distal-to-proximal trajectory under continuous ultrasound visualization. Small aliquots of injectate are first delivered to hydro-localize the tip and confirm the intended plane between the deep surface of the TCL and the underlying subsynovial connective tissue. Progressive HD is then performed to separate the ligament from the underlying tissues while avoiding excessive injection pressure. This initial step is intended to establish and expand the deep working plane beneath the TCL and does not constitute a TCL release. Without changing the skin entry point, the same trajectory is used to continue HD around the median nerve. Injectate is directed to achieve circumferential perineural separation, including both the superficial and deep aspects of the nerve, while avoiding intraneural injection. This workflow sequentially addresses the TCL undersurface and the perineural plane surrounding the median nerve through a single continuously visualized in-plane pathway. The real-time injection technique is demonstrated in Figure 2 and Video 1.

**Video 1:** Real-time ultrasound-guided single-entry dual-target hydrodissection technique for carpal tunnel syndrome The video begins at the first carpometacarpal (1st CMC) joint, with the cortical surface of the first metacarpal maintained in plane, and demonstrates medial transducer translation across the carpal tunnel while preserving the same scanning plane. After this tracing step, the needle is advanced along an oblique distal-to-proximal in-plane trajectory. The sequence demonstrates the maintained scanning plane, single-entry needle pathway, and injectate delivery during the procedure

Final confirmation and immediate assessment

After completion of HD, a final confirmation sweep is performed along the maintained scanning plane across the carpal tunnel to verify continuity of the fluid plane along the TCL undersurface, fluid surrounding the median nerve consistent with perineural halo formation, and the absence of hematoma or abnormal Doppler signals. Representative post-procedure distal-to-proximal confirmation scanning is demonstrated in Video 2.

**Video 2:** Distal-to-proximal post-hydrodissection confirmation scan demonstrating perineural halo formation around the median nerve Following completion of the single-entry, dual-target hydrodissection technique, the transducer is translated in a distal-to-proximal manner along the maintained scanning plane across the carpal tunnel. The video demonstrates fluid surrounding the median nerve on its superficial and deep aspects, consistent with perineural halo formation

Cadaveric anatomic validation

**Figure 3:**
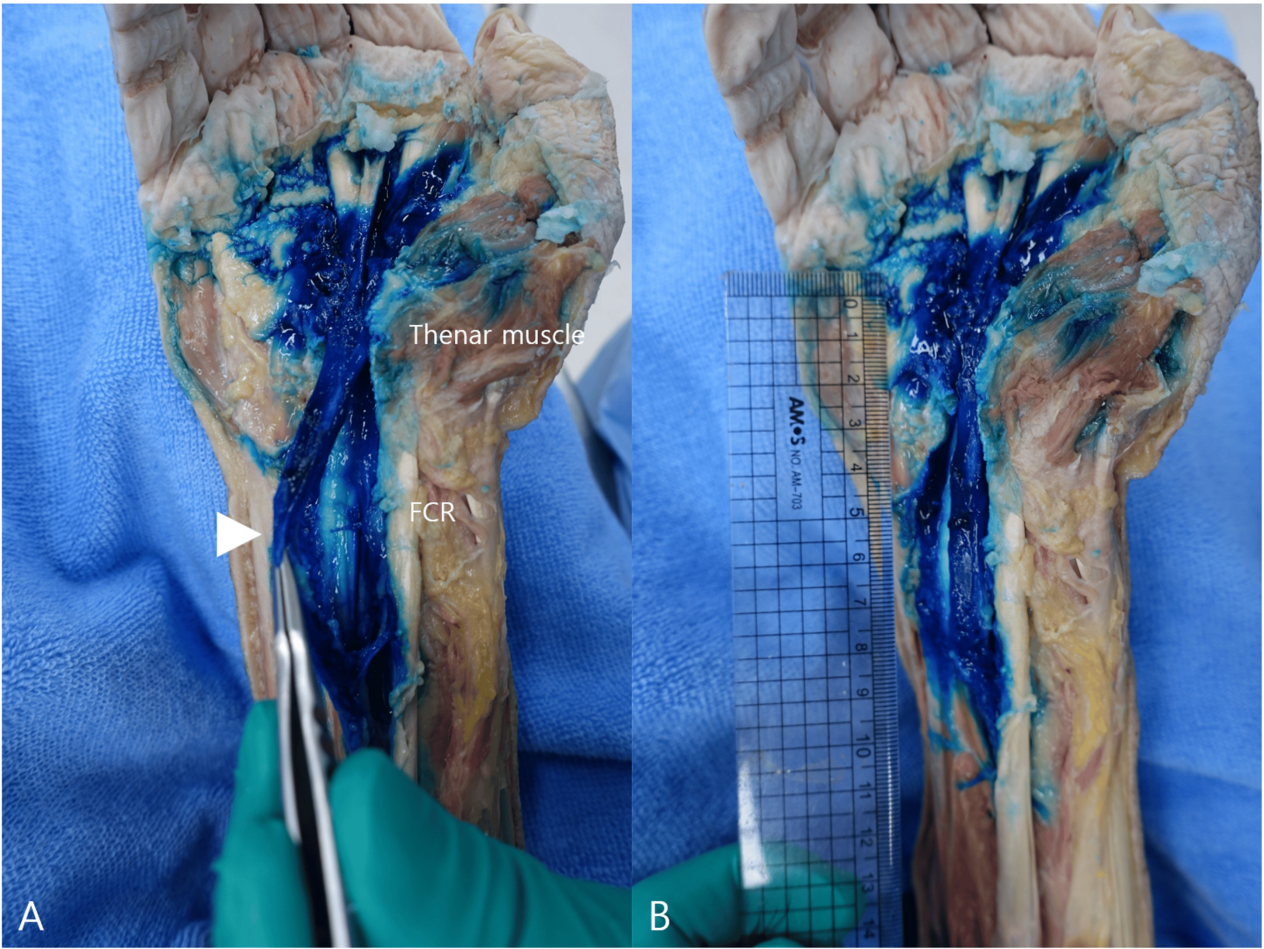
Cadaveric validation of injectate distribution following the described single-entry dual-target hydrodissection technique (A) Cadaveric dissection demonstrates methylene blue surrounding the median nerve on both its superficial and deep aspects. The median nerve (white arrowhead) is identified and gently elevated with forceps, confirming dye distribution both above and below the nerve, consistent with circumferential perineural halo formation. The flexor carpi radialis (FCR) region remained relatively unstained, suggesting that dye distribution was concentrated within the intended hydrodissection plane rather than diffusely involving the adjacent FCR compartment. (B) Cadaveric dissection demonstrates proximal longitudinal dye spread extending from the needle entry point to approximately 12 cm proximally, corresponding to about 8 cm proximal to the wrist crease, as documented with a ruler

To assess the anatomical plausibility of the proposed technique, the same procedural approach was performed in one fresh-frozen cadaver using both wrists. Under ultrasound guidance, 10 mL of methylene blue was injected per wrist using a 23-gauge, 6-cm needle, followed by dissection 60 minutes after injection. Cadaveric dissection demonstrated methylene blue surrounding the median nerve on both its superficial and deep aspects. The median nerve was identified and gently elevated with forceps, confirming dye distribution both above and below the nerve, consistent with circumferential perineural halo formation. The adjacent flexor carpi radialis (FCR) region remained relatively unstained, suggesting that dye distribution was concentrated within the intended hydrodissection plane rather than diffusely involving the adjacent FCR compartment. In addition, longitudinal dye propagation was observed extending proximally from the needle entry point to approximately 12 cm, corresponding to about 8 cm proximal to the wrist crease. These findings support continuity of the intended HD plane and provide an anatomic correlate for the proposed single-entry dual-target strategy (Figure 3). The cadaveric validation findings are summarized in Table 2.

**Table 2:** Structured summary of cadaveric validation findings

Parameter	Finding
Cadaver specimens	1 fresh-frozen cadaver
Wrists evaluated	2 wrists
Injectate	Methylene blue
Injectate volume	10 mL per wrist
Needle	23-gauge, 6-cm needle
Dissection timing	60 minutes after injection
Superficial dye spread around the median nerve	Observed bilaterally
Deep dye spread around the median nerve	Observed bilaterally
Circumferential perineural halo pattern	Supported bilaterally
Proximal longitudinal dye extension	Approximately 12 cm from the entry point
Relative proximal extension	Approximately 8 cm proximal to the wrist crease
Interpretation	Descriptive anatomic validation of the intended hydrodissection plane

Because both wrists originated from a single cadaver specimen, these findings should be interpreted as descriptive anatomic validation only and not as evidence of reproducibility, comparative superiority, or clinical efficacy.

Post-procedure care and follow-up

A simple dressing is applied after the procedure. Patients are advised to modify activity for 24-48 hours and are educated regarding warning signs such as increasing pain, swelling, or new neurologic deficit. Clinical outcomes were not evaluated in the present technical report.

Safety considerations and risk mitigation

This technique integrates diagnostic ultrasound assessment with targeted intervention, allowing procedural planning to be adjusted according to the real-time location of the compression zone and the presence of anatomic variants. Potential complications include vascular injury, tendon puncture, nerve irritation, intraneural injection, and infection. Particular care should be taken to avoid the palmar cutaneous branch of the median nerve, radial-sided vascular structures, and the recurrent motor branch. If the pre-procedural safety scan identifies a persistent median artery or a bifid median nerve, the needle trajectory should be adjusted slightly radially or ulnarly, or the procedure should be reconsidered if a safe path cannot be established. Risk mitigation strategies include a systematic pre-scan, continuous needle tip visualization, hydro-localization before full injectate delivery, and low-pressure injection throughout the procedure.

## Discussion

This technical note describes a single-entry dual-target ultrasound-guided HD technique for CTS based on a standardized scanning approach initiated at the 1st CMC joint, maintained during medial transducer translation across the carpal tunnel, and combined with an oblique distal-to-proximal in-plane needle trajectory. The conceptual aim of this approach is to sequentially address two clinically relevant tissue planes through a single continuously visualized pathway: first, the deep working plane beneath the TCL, and second, the circumferential perineural plane surrounding the median nerve. Importantly, HD along the undersurface of the TCL in this context is intended to establish and expand the deep working plane beneath the ligament and does not constitute TCL release.

The rationale for this workflow is that CTS is increasingly understood as a multifactorial entrapment neuropathy involving not only focal compression of the median nerve, but also TCL-related mechanical constraint, subsynovial fibrosis, perineural adhesions, and impaired neural gliding [3,4]. From this perspective, a technique that sequentially addresses the deep TCL interface and the perineural plane may provide a practical framework for HD in selected patients. The present method was not designed to demonstrate superiority over existing techniques, but rather to describe a structured procedural strategy intended to simplify workflow while maintaining continuous in-plane needle visualization.

A practical challenge in conventional ultrasound-guided HD is that achieving circumferential perineural spread around the median nerve may require repeated needle repositioning, multiple skin entries, or a combination of short-axis and long-axis maneuvers [1,2,4,5]. Although these approaches may be effective, they can increase procedural complexity and technical demand. In contrast, the present technique uses a maintained 1st CMC-based scanning plane established during medial transducer translation and a sequential single-entry workflow to reach both the TCL undersurface and the perineural plane through one oblique in-plane trajectory. The conceptual and technical distinctions between this workflow and commonly used ultrasound-guided CTS HD approaches are summarized in Table 3. This table is intended as a descriptive comparison of procedural concepts rather than as evidence of comparative clinical advantage.

**Table 3:** Comparison of ultrasound-guided HD approaches for CTS HD: hydrodissection; CTS: carpal tunnel syndrome

Feature	Present technique	Conventional short-axis approach	Conventional long-axis approach
Direction of advancement	Distal-to-proximal	Variable	Usually distal-to-proximal or proximal-to-distal
Needle trajectory	Oblique in-plane	Transverse in-plane	Longitudinal in-plane
Working concept	Single-entry dual-target	Primarily perineural	Primarily longitudinal perineural
Need for redirection/repositioning	Minimized by a structured single-entry workflow	Often variable depending on halo formation	May require complementary redirection for circumferential spread

Another strength of the present report is the inclusion of cadaveric anatomic validation. Using the same procedural approach in one fresh-frozen cadaver with bilateral wrist injections, methylene blue dissection demonstrated dye surrounding the median nerve on both its superficial and deep aspects, consistent with circumferential perineural halo formation. The adjacent FCR region remained relatively unstained, suggesting that injectate distribution was concentrated within the intended hydrodissection plane rather than diffusely involving the adjacent FCR compartment. In addition, proximal longitudinal dye propagation was observed extending from the needle entry point to approximately 12 cm, corresponding to about 8 cm proximal to the wrist crease. These findings support continuity of the intended HD plane and provide an anatomic correlate for the proposed strategy. However, because both wrists originated from a single cadaver specimen, these observations should be interpreted strictly as descriptive evidence of anatomical plausibility rather than as evidence of reproducibility, comparative superiority, or clinical efficacy.

The present technique may offer several practical procedural advantages. By maintaining a continuous in-plane view within a structured scanning corridor, it may help reduce unnecessary needle redirection and preserve anatomical orientation throughout the procedure. The mandatory pre-procedural safety scan may also assist the operator in identifying anatomic variants, including a bifid median nerve, persistent median artery, tenosynovitis, or space-occupying lesions, before needle advancement. In this sense, the technique integrates diagnostic ultrasonographic assessment with intervention planning rather than treating injection as an isolated procedural step. Nevertheless, these potential advantages remain conceptual and were not directly tested in the present report.

This report should be interpreted in light of several limitations. First, it is a technical description supported by cadaveric dye validation, not a prospective clinical outcome study. No conclusions can therefore be drawn regarding superiority, durability, or comparative efficacy relative to other ultrasound-guided CTS techniques. Second, the cadaveric component involved only one fresh-frozen cadaver with bilateral wrist injections, and the two wrists cannot be considered independent observations. Third, the cadaveric findings were qualitative; no volumetric analysis, graded spread assessment, or comparator technique was included. Although no volumetric or graded spread analysis was performed, simple descriptive procedural and distribution metrics were added to improve clarity of reporting. Fourth, the cadaveric injectate consisted of methylene blue, whereas the clinical technique was described using 5% dextrose in water, and the distribution characteristics of different injectates may not be identical [6]. Fifth, procedural safety and technical success remain operator dependent despite the use of a structured scanning plane.

Future studies should evaluate this technique in prospective feasibility cohorts and comparative clinical studies against established short-axis and long-axis HD approaches. Standardized reporting should include injectate composition and volume, sonographic endpoints, procedural time, complications, patient-reported outcomes, and interval changes in median nerve cross-sectional area [6]. Beyond CTS, the broader value of this approach may lie in its potential applicability to other anatomically constrained regions [7]. By simplifying circumferential HD through a single-entry structured in-plane workflow, this technique may provide a transferable framework for anatomically guided interventions at other sites. Additional cadaveric studies using more specimens would also be helpful to determine the consistency of injectate distribution across different wrists and anatomical variants.

## Conclusions

This technical note describes a novel ultrasound-guided single-entry dual-target HD technique for CTS using a standardized scanning approach initiated at the 1st CMC joint and maintained during medial transducer translation across the carpal tunnel, combined with a continuous oblique distal-to-proximal in-plane needle trajectory. This approach is intended to provide a structured procedural framework for HD while maintaining continuous needle visualization. Cadaveric methylene blue dissection in one fresh-frozen cadaver using both wrists supported the anatomical plausibility of the intended injectate distribution. However, these findings should be interpreted as descriptive anatomic validation only and not as evidence of clinical efficacy, comparative superiority, reproducibility, or procedural safety advantage. Further prospective and comparative studies are needed to define the clinical role of this approach relative to existing ultrasound-guided CTS HD techniques.
